# The Midwifery Muddle

**Published:** 1895-02-23

**Authors:** 


### THE MIDWIFERY MUDDLE.

The hopeless muddle in which the midwifery question now lies was well illustrated by the proceedings of the Birmingham and Midland Counties Branch of the British Medical Association last month. The committee appointed to consider the various schemes proposed in connection with the registration of midwives reported that midwifery nurses should be trained, examined, and registered, under the supervision of the county councils, and that they should be strictly confined to attendance on natural labours. To this Sir Walter Foster moved as an amendment that, "inasmuch as the proper care of these lives [*i.e.*, of parturient women] before, during, and after the act of labour, involves a knowledge of almost the whole arts of medicine and surgery, no one should be entrusted with them save fully-trained and qualified practitioners." We congratulate Sir Walter Foster on occupying a logically unassailable position; nevertheless, his amendment was lost: but, then, so also was the original motion, and the question remains *in statu quo*. If the medical profession will support Sir Walter Foster and will persuade the Government to provide for the expense of supplying every woman with qualified medical attendance, male or female, at the time of her confinement, well and good. If, however, they decline, or are unable to do that, the continued refusal to protect these women, by enforcing the education and registration of midwives, will remind many of the fable of the dog in the manger.

